# Tumor-like cortical venous thrombosis: Case report and literature review

**DOI:** 10.1097/MD.0000000000047866

**Published:** 2026-02-28

**Authors:** Lan Cheng, Lianjie Li

**Affiliations:** a Department of Cardiovascular Medicine, Ningbo No. 2 Hospital, Ningbo, P.R. China; b Department of Neurosurgery, Ningbo No. 2 Hospital, Ningbo, P.R. China.

**Keywords:** brain tumor, case report, cortical hemorrhage, cortical venous thrombosis, magnetic resonance imaging, ring reinforcement

## Abstract

**Rationale:**

Isolated cortical venous thrombosis (ICoVT) is a rare type of cerebral venous thrombosis. Because of its atypical clinical presentation and imaging features that resemble those of brain tumors, ICoVT is often misdiagnosed as a brain tumor.

**Patient concerns:**

This study reports 2 patients with ICoVT. Both experienced a gradual onset of symptoms—one presented primarily with headache, whereas the other developed progressive limb weakness.

**Diagnoses and interventions:**

Brain magnetic resonance imaging revealed a large hemorrhagic lesion in 1 patient, showing ring enhancement and extensive surrounding edema. The other patient had an ischemic lesion with similar ring enhancement and marked perilesional edema.

**Outcomes:**

Given the suspicion of a brain tumor, both patients underwent surgical biopsy. Intraoperatively, cortical venous thrombosis was identified, and histopathological findings confirmed the diagnosis of ICoVT. Both patients subsequently received effective anticoagulant therapy.

**Lessons:**

ICoVT has atypical clinical and imaging characteristics and is easily mistaken for brain tumors. When neuroimaging demonstrates cortical hemorrhage or ischemic lesions accompanied by extensive edema, the possibility of ICoVT should be considered.

## 1. Introduction

Isolated cortical venous thrombosis (ICoVT) refers to thrombosis occurring exclusively within the cerebral cortical veins.^[1]^ ICoVT represents a distinct form of cerebral venous thrombosis (CVT) and is relatively uncommon in clinical practice. Because its clinical manifestations are often atypical, ICoVT can be difficult to identify using current imaging techniques. The parenchymal changes associated with CVT are typically variable, nonspecific, and may appear as subarachnoid hemorrhage, cerebral edema, infarction, or hemorrhage.^[2–6]^ When cerebral infarction or hemorrhage coexists, cortical venous thrombosis may closely mimic a brain tumor on imaging. The mechanisms underlying ICoVT are susceptible to “tumor-like” necrosis, edema, and enhancement. These phenomena may be associated with an inflammatory factor storm, disruption of the blood-brain barrier, and inflammatory necrosis. We described 2 patients whose clinical and radiological findings were initially suggestive of glioma but were later confirmed as ICoVT based on surgical pathology in this report.

## 2. Case presentation

This study received approval from the Ethics Committee of Ningbo No. 2 Hospital. Written informed consent for the publication of these 2 case reports and the accompanying images was obtained from the patients and their family members. The case report was exempted from Institutional Review Board review.

### 2.1. Case 1

A 51-year-old woman was admitted to the hospital on March 8, 2020 with a history of recurrent headache for 2 months, worsening over the previous 10 days. The headache was described as a persistent dull pain localized to the left temporal region, accompanied by nausea and vomiting but without fever, photophobia, or limb convulsions. She was otherwise healthy. Physical examination findings were unremarkable.

**Figure 1.**
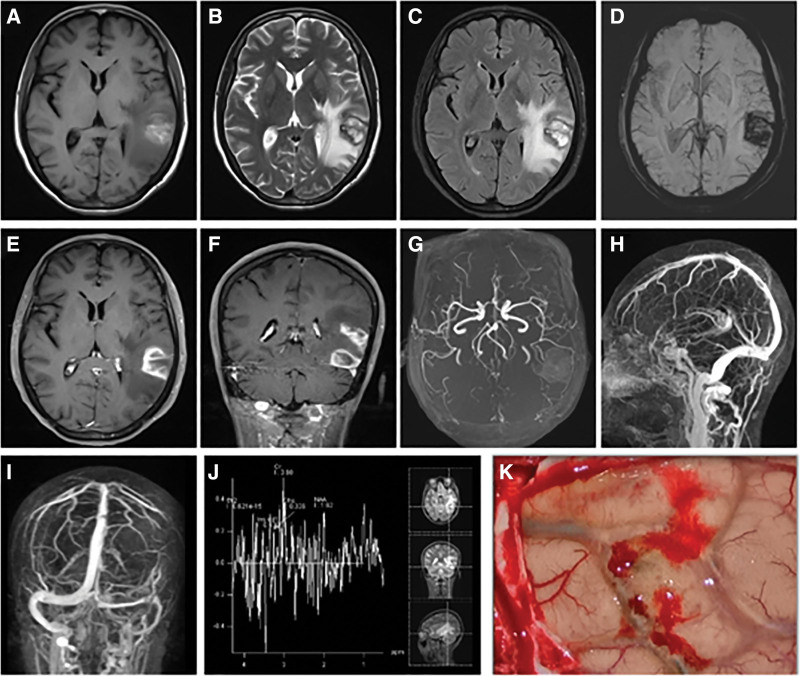
Preoperative imaging and intraoperative findings in patient 1. (A) Axial MRI T1-weighted image showing a lesion in the left temporal lobe cortex with heterogeneous mixed signals and focal high signal intensity. (B) Axial MRI T2-weighted image demonstrating a ring-shaped low signal surrounding the lesion, irregular high signals internally, and associated white matter edema. (C) Axial MRI FLAIR sequence showing irregular internal high signals and perilesional white matter edema. (D) Axial MRI SWI sequence revealing low signal intensity within the lesion. (E, F) Axial and sagittal contrast-enhanced MRI showing 2 mass-like abnormal signals in the left temporal lobe with ring-shaped enhancement, measuring 2.4 × 2.3 × 1.8 cm and 2.3 × 2.1 × 1.5 cm, respectively. (G–I) MRA and MRV showing no significant abnormalities in intracranial vessels. (J) MRS demonstrating a markedly elevated choline (Cho) peak and decreased N-acetyl aspartate (NAA) peak, with a Cho/NAA ratio of 4.2. (K) Intraoperative findings showing swollen, yellowish cortical tissue with cortical venous thrombosis (arrows). MRA = magnetic resonance angiography, MRI = magnetic resonance imaging, MRS = magnetic resonance spectroscopy, MRV = magnetic resonance venography.

Magnetic resonance imaging (MRI) of the brain with contrast enhancement, magnetic resonance angiography, magnetic resonance venography (MRV), susceptibility-weighted imaging (SWI), and MR spectroscopy (MRS) revealed 2 mass-like lesions in the left temporal lobe, measuring 2.4 × 2.3 × 1.8 cm and 2.3 × 2.1 × 1.5 cm, respectively. The lesions demonstrated high signal intensity on T1-weighted images and mixed high signal on T2-weighted images (T2WI). SWI showed multiple foci of signal loss within the lesions, while MRS revealed a marked increase in the choline peak and a decrease in the N-acetyl aspartate peak within selected regions of interest. The enhanced MRI sequences demonstrated prominent ring-like enhancement. Magnetic resonance angiography and MRV findings showed no apparent abnormalities in the intracranial vasculature (Fig. 1A–J).

**Figure 2.**
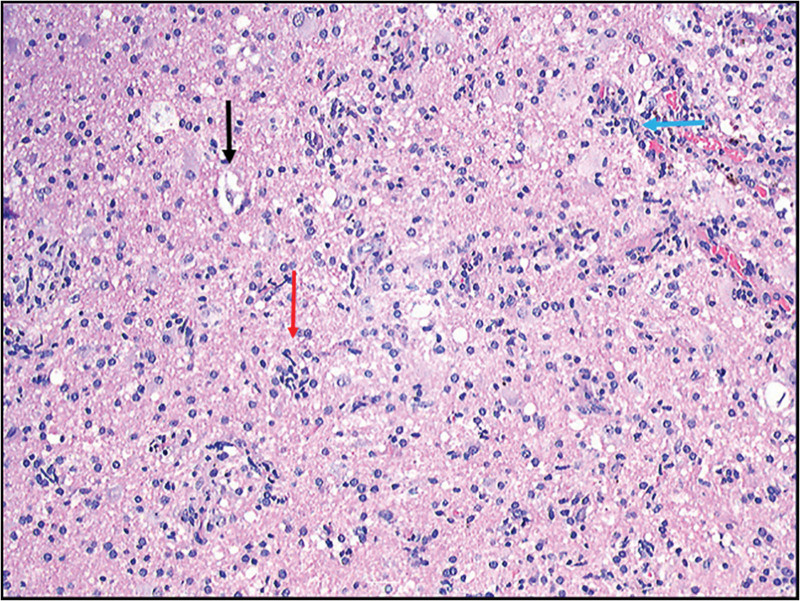
Haematoxylin and eosin (HE) staining of case 1 (magnification 40×) reveals focal encephalomalacia, accompanied by gliosis (denoted by the red arrow), lymphocytic infiltration (denoted by the blue arrow), and foam cell infiltration surrounding blood vessels (denoted by the black arrow), indicative of an infarctive lesion.

A provisional diagnosis of hemorrhagic glioma was made, and craniotomy was performed for resection. Intraoperatively, the affected brain tissue exhibited edema and yellow discoloration, with evidence of cortical venous thrombosis and a chronic subcortical hematoma (Fig. 1K). The frozen-section histopathological analysis revealed localized encephalomalacia, glial cell proliferation, and perivascular infiltration by lymphocytes, plasma cells, and foam cells. Notably, no tumor-like lesions were detected (Fig. 2). Immunohistochemistry revealed focal myelin loss, foam cells, and infiltration by T lymphocytes, but no neoplastic cells were detected within the lesion. Postoperatively, low-molecular-weight heparin sodium (5000 IU) was administered subcutaneously twice daily for 2 weeks. The patient’s headache subsequently improved, and after discharge, warfarin 2.5 mg was taken orally once daily for 6 months. During 1 year of follow-up, the headache did not recur.

### 2.2. Case 2

A 75-year-old man was admitted to the hospital on May 24, 2020 with progressive weakness of the left limb over the preceding 3 months. Throughout the illness, he did not experience nausea, vomiting, photophobia, headache, seizures, or loss of consciousness. Three months earlier, he had been diagnosed with bilateral pneumonia following an episode of high fever. He had no history of diabetes, hypertension, or autoimmune disease.

Neurological examination revealed grade IV muscle strength in the left upper limb and grade II in the left lower limb. Light-touch and pain sensation in the left limb were diminished compared with the contralateral side, and the left Babinski sign was positive.

**Figure 3.**
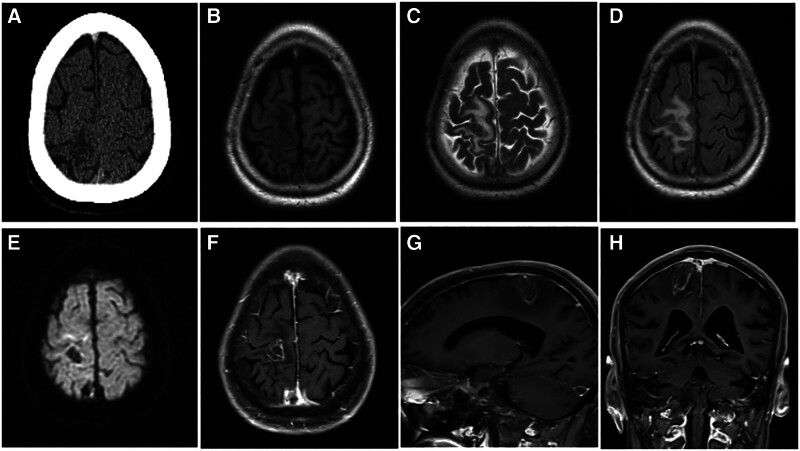
Preoperative neuroimaging findings of patient 2. (A) A non-contrast computed tomography (CT) scan of the brain in the axial plane reveals a patchy low-density area measuring approximately 1.9 cm × 1.6 cm × 1.2 cm in the right central region. (B) Axial T1-weighted magnetic resonance imaging (MRI) indicates the presence of the lesion in the right central cortical area, characterized by a localized low signal. (C, D) Axial T2-weighted MRI and fluid-attenuated inversion recovery (FLAIR) sequences demonstrate the lesion with a slightly low signal, accompanied by irregular high signal intensity surrounding the lesion, indicative of edema in the adjacent white matter. (E) Axial diffusion-weighted imaging (DWI) shows the lesion with a low signal. (F–H) Post-contrast MRI of the brain in axial, sagittal, and coronal planes reveals the lesion exhibiting ring enhancement.

Cranial computed tomography demonstrated a patchy low-density area approximately 1.9 × 1.6 × 1.2 cm in the right central region (Fig. 3A). Contrast-enhanced brain MRI showed a nodular lesion in the same region with a well-defined margin, low signal on T1-weighted images, high signal on T2WI, low signal on diffusion-weighted imaging, and surrounding patchy edema. The post-contrast sequences demonstrated ring-shaped enhancement of the lesion (Fig. 3B–H). Serum tumor-marker levels were within normal limits.

**Figure 4.**
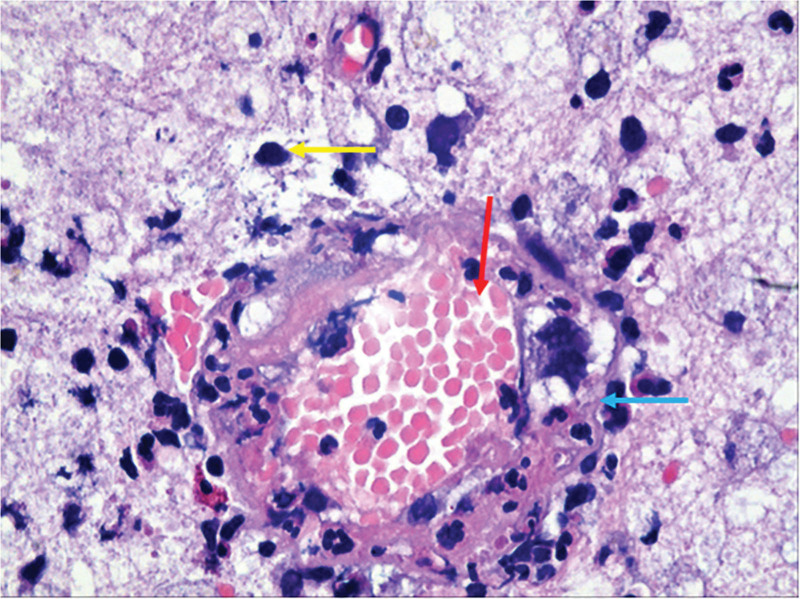
Haematoxylin and eosin (HE) staining of case 2 at 100× magnification reveals the cerebral cortex veins and the brain tissue in the infarction area. The blue arrow indicates the boundary of the cortical vein, whereas the red arrow highlights the presence of erythrocytes within the vascular lumen. Additionally, the yellow arrow denotes the accumulation of iron within the foam cells located in the infarcted region.

A provisional diagnosis of glioma in the right central region was made, and craniotomy for resection was planned. Intraoperatively, marked cerebral edema, gray–white discoloration of the lesion, and venous thrombosis within the central sulcus were observed. Postoperative histopathological examination revealed focal cerebral infarction with dense infiltration of foam cells within the infarcted region. Iron-hydroxide deposition was identified within these foam cells, and no neoplastic lesions were found (Fig. 4). Low-molecular-weight heparin sodium (5000 IU) was administered subcutaneously twice daily for 2 weeks, after which the patient was transitioned to oral warfarin (2.5 mg) once daily for 3 months. The patient was followed for 1 year. Postoperatively, muscle strength in the left upper limb improved to grade V, and that in the left lower limb improved to grade IV.

## 3. Discussion

CVT accounts for 1% to 2% of all stroke cases,^[7]^ and encompasses cranial venous-sinus thrombosis, ICoVT, and deep cerebral venous thrombosis. ICoVT represents approximately 6% of all CVT.^[7]^ Headache and focal neurological deficits—such as sensory loss, aphasia, hemiplegia, and alexia—are the most common clinical manifestations. Moreover, ICoVT may precipitate focal seizures.^[8]^ A minority of patients develop signs of raised intracranial pressure; however, these are nonspecific.

This study describes 2 patients who presented with headache and focal neurological deficits. The shared imaging characteristic was a tumor-like venous infarction pattern. One patient exhibited multiple subcortical temporal hemorrhages, whereas the other demonstrated subcortical infarction in the central region. Both were initially misdiagnosed preoperatively as gliomas, but surgical pathology confirmed cortical venous thrombosis with associated cerebral infarction and hemorrhage. Additionally, we elucidate the similarities and differences in the ring enhancement patterns among cortical venous thrombosis, microhemorrhage, high-grade glioma, and metastasis, as detailed in Table 1. The etiology of ICoVT is multifactorial. Several studies have identified risk factors that can precipitate or contribute to CVT; however, in some patients, the cause remains undetermined in clinical practice. The underlying causes of CVT and ICoVT are thought to be largely similar. Reported risk factors for ICoVT include oral contraceptive use, pregnancy or the puerperium, malignant tumors, hereditary thrombophilia, infections involving the ear, nose, or throat, cranial trauma or surgery, prior lumbar puncture, systemic infection, low intracranial pressure, and certain medications.^[9–13]^ Previous studies have reported instances of cerebral venous-sinus thrombosis (CVST) associated with COVID-19 infection.^[14]^ The primary mechanisms implicated in CVST include a hypercoagulable state, vascular endothelial dysfunction, and altered flow dynamics.^[15]^ Anatomically, the cortical veins have thin walls that lack muscular and elastic fibers, contractility, and valves, predisposing them to venous reflux and blood stasis. When these structural characteristics are coupled with increased blood viscosity and venous endothelial injury, the resulting hemodynamic disturbance facilitates thrombosis within the cortical veins. In one of the present cases, the etiology of ICoVT was unknown, whereas in the other, the patient had a recent history of high fever and pneumonia, suggesting damages of vascular endothelial cells caused by the inflammatory factor storm and systemic inflammation.

**Table 1:** Differential diagnosis of ring enhancement in cortical venous thrombosis, microhemorrhage, high-grade glioma, and metastasis.

Feature	Cortical venous thrombosis	Microhemorrhage	High-grade glioma	Metastasis
Enhancement pattern	Irregular, linear, or punctate enhancement along the venous course	No enhancement or blurred ring enhancement (resorption phase)	Rosette-like enhancement with unevenly thickened walls and irregular inner margins	Round or oval ring enhancement with unevenly thickened walls and irregular inner margins
DWI findings	Diffusion restriction (less prominent than in abscess)	Nonspecific changes (resorption phase)	Nonrestricted diffusion in the necrotic core (low signal, high ADC value)	Nonrestricted diffusion in the necrotic core (low signal, high ADC value)
Perfusion imaging (CBV)	Normal or reduced	Nonspecific changes	Increased	Normal or reduced
Clinical clues	Headache, seizures, or focal neurological deficits; history and lab tests (e.g., d-dimer) needed	History of trauma or hypertension; history and lab tests needed	Rapid progression, significant edema, mass effect; molecular markers (e.g., IDH mutation, MGMT methylation) required	History of primary malignancy; systemic workup (e.g., PET-CT) needed
Other imaging features	Follows venous anatomy	Resorption phase, significant perilesional edema	Irregular tumor margins (“crab-like” appearance)	Multiple lesions with significant perilesional edema

CBV = cerebral blood volume.

The diagnosis of ICoVT cannot rely solely on clinical manifestations. Characteristic imaging findings include the “linear sign” of the obstructed cortical vein, which serves as a direct indicator of venous occlusion.^[14]^ Other notable features encompass focal edema, ischemic infarction, subarachnoid hemorrhage, hemorrhagic infarction, and lobar hemorrhage, all resulting from venous outflow obstruction. Non-visualization of the thrombosed cortical vein on digital subtraction angiography is another diagnostic feature.^[5]^ Because cortical veins vary in number, caliber, and drainage pattern, imaging appearances can be inconsistent and may not always yield a definitive diagnosis. Moreover, the distribution of cortical or subcortical lesions in ICoVT differs from that of arterial infarctions, with necrosis usually less extensive. In this study, we have identified and delineated the key distinguishing features among cortical venous thrombosis, demyelinating lesions, encephalitis, and cerebral infarction, as presented in Table 2. Recognition of these distinctions provides valuable clues for the differential diagnosis of ICoVT.^[16]^

**Table 2:** Key differentiation points between cortical venous thrombosis, demyelinating lesions, encephalitis, and cerebral infarction.

Differentiation points	Cortical venous thrombosis	Demyelinating lesions	Encephalitis	Cerebral infarction
Etiology	Infection, hypercoagulable state, impaired venous return	Autoimmune abnormalities, genetic factors	Viral, bacterial, fungal infections	Atherosclerosis, embolism, small vessel disease
Imaging findings	Ovoid or round lesions in cortical and subcortical areas prone to hemorrhage, low signal on T2WI enhanced gyral or patchy enhancement	Elliptical T2 hyperintense lesions near cortex and around ventricles, segmental enhancing lesions in spinal cord	Diffuse or focal abnormal signals in brain parenchyma, meningeal enhancement	Low-density lesions in arterial territory (CT) or long T1 long T2 signals Restricted diffusion
Clinical features	Headache, seizures, focal neurological deficits	Multifocal neurological dysfunction	Fever, headache, impaired consciousness, inflammatory changes in cerebrospinal fluid	Sudden focal neurological deficits symptoms consistent with arterial territory
Laboratory tests	Elevated d-dimer, infection indicators may be abnormal	Positive autoantibodies—oligoclonal bands in cerebrospinal fluid may be positive	Increased white blood cells and protein in cerebrospinal fluid, positive pathogen detection	Abnormal vascular risk factors cerebrospinal fluid usually normal
Treatment approach	Anticoagulation therapy control infection, reduce intracranial pressure	Immunotherapy, symptomatic treatment	The use of antibiotics, immune modulation	Thrombolysis antiplatelet, lipid-lowering, and other secondary prevention measures

In the 2 patients described in this study, the disease course was prolonged, and persistent venous hypertension impaired cerebral perfusion pressure, leading to vascular and cytotoxic edema followed by neuronal injury and cell death. Neuroimaging in patients with ICoVT may show cerebral infarction, intracerebral hemorrhage, parenchymal edema, or necrosis—features that closely resemble those of brain tumors and therefore require careful differentiation. When imaging reveals isolated cortical infarction or hemorrhagic lesions accompanied by marked edema, ICoVT should be considered in the differential diagnosis.

The principal pathological features of ICoVT include focal necrosis, hemorrhage, and dilation of small cortical veins containing thrombi. Microscopically, small vessels demonstrate neuronal degeneration, phagocytosis, gliosis, and endothelial proliferation.^[17]^ In the present cases, patient 1 exhibited focal necrosis and gliosis, whereas patient 2 showed pronounced cortical venous thrombosis, findings consistent with the recognized pathological spectrum of CVT. Although routine biopsy and pathological examination are not generally recommended for ICoVT, in patients without characteristic imaging findings—particularly when differentiation from a brain tumor is difficult—early diagnosis and treatment are key to a favorable outcome. In such cases, pathological examination is diagnostically valuable, and surgical biopsy may be necessary to achieve a definitive diagnosis.

Management of ICoVT primarily involves anticoagulation with heparin once the clinical diagnosis is established.^[1,6,18]^ Intravenous or subcutaneous heparin is typically administered for approximately 2 weeks, followed by oral warfarin therapy, even when hemorrhagic infarction is present. Antiepileptic drugs should be initiated in patients who develop seizures. For patients with cerebral edema or raised intracranial pressure, osmotic dehydration and intracranial pressure–lowering measures are indicated. In cases of refractory intracranial hypertension, decompressive craniectomy may be considered when clinically necessary.

In conclusion, we report 2 cases of ICoVT that were initially misdiagnosed as gliomas on preoperative imaging but were subsequently confirmed by surgical biopsy and histopathology. Both patients achieved favorable outcomes with timely anticoagulation therapy. In the imaging evaluation of a solitary cortical hemorrhage or infarction with significant edema, it is crucial to include intra-CVT in the differential diagnosis. This consideration remains pertinent even when MRS suggests the presence of a neoplasm, indicated by elevated choline/N-acetyl aspartate ratios. Therefore, the use of MRV or SWI should be considered for further assessment.

## Acknowledgments

We would also like to thank Editage (www.editage.cn) for English language editing.

## Author contributions

**Funding acquisition:** Lan Cheng.

**Writing – original draft:** Lianjie Li.

**Writing – review & editing:** Lianjie Li.
